# Chronic Myeloid Leukemia: A Model Disease of the Past, Present and Future

**DOI:** 10.3390/cells10010117

**Published:** 2021-01-10

**Authors:** Valentina R. Minciacchi, Rahul Kumar, Daniela S. Krause

**Affiliations:** 1 Georg-Speyer-Haus, Institute for Tumor Biology and Experimental Therapy, Paul-Ehrlich-Str. 42-44, 60596 Frankfurt am Main, Germany; Minciacchi@gsh.uni-frankfurt.de (V.R.M.); kumar@gsh.uni-frankfurt.de (R.K.); 2 German Cancer Research Center (DKFZ), D-69120 Heidelberg, Germany; 3 German Cancer Consortium (DKTK), Im Neuenheimer Feld 280, D-69120 Heidelberg, Germany; 4 Frankfurt Cancer Institute, 60596 Frankfurt, Germany; 5 Faculty of Medicine, Medical Clinic II, Johann Wolfgang Goethe University, 60596 Frankfurt, Germany

**Keywords:** chronic myeloid leukemia, history, targeted therapy, metabolism, bone marrow microenvironment, combination therapies

## Abstract

Chronic myeloid leukemia (CML) has been a “model disease” with a long history. Beginning with the first discovery of leukemia and the description of the Philadelphia Chromosome and ending with the current goal of achieving treatment-free remission after targeted therapies, we describe here the journey of CML, focusing on molecular pathways relating to signaling, metabolism and the bone marrow microenvironment. We highlight current strategies for combination therapies aimed at eradicating the CML stem cell; hopefully the final destination of this long voyage.

## 1. The History of Chronic Myeloid Leukemia

**Figure 1:**
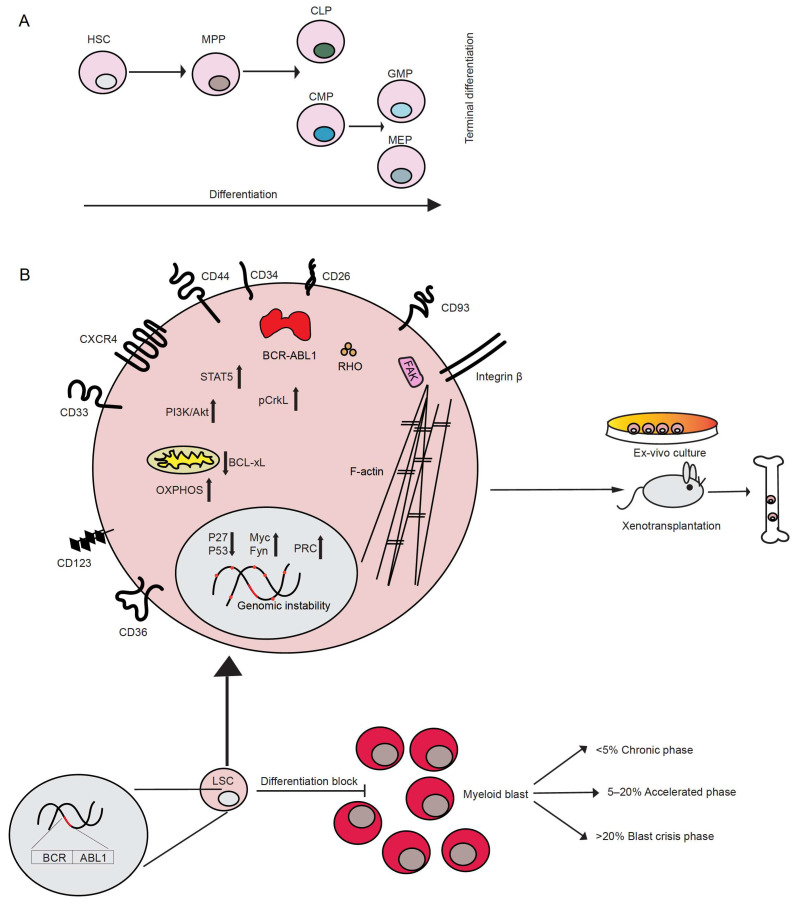
(**A**) Schematic depicting the hierarchy of normal hematopoiesis inside the bone marrow. Hematopoietic stem cells reside at the top of the hierarchy. During differentiation, cells subsequently commit to different lineages ending in their terminal differentiation. The cells of the normal hematopoietic lineage are shown as circles with differentially colored nuclei representing different maturation states or lineages. (**B**) Diagram illustrating the characteristics, properties and signaling pathways following the transformation of hematopoietic stem cells to chronic myeloid leukemia (CML) stem cells. The smaller circle at the bottom shows the LSC and above is the magnified image of the LSC showing the relevant cell surface receptors, components of the cytoskeleton (elongated black lines—F-actin), cytoplasmic adaptor proteins, mitochondria (yellow), and transcription factors associated with CML stem cells are also shown (in a gray circle representing the nucleus inside the LSC). HSC = hematopoietic stem cell; MPP = multipotent progenitor cell, CLP = common lymphoid progenitor cell; CMP = common myeloid progenitor cell; GMP = granulocyte-macrophage progenitor cell; MEP = megakaryocyte-erythrocyte progenitor cell; LSC = leukemic stem cell; PI3K = phosphoinositide 3-kinase; PRC = polycomb receptor complex; CXCR4 = C-X-C chemokine receptor type 4; STAT5 = signal transducer and activator of transcription 5.

The first descriptions of chronic myeloid leukemia (CML), or as it was called at the beginning, chronic granulocytic leukemia, can be dated back to 1845. At that time, almost simultaneously, two independent pathologists, John Bennett and Rudolf Virchow, published case reports of patients with splenomegaly, an enlarged liver and leukocytosis [1,2]. However, little, if anything, was known about the disease. It was Virchow himself who first coined the term “Leukämie”, meaning “white blood”, to indicate the idea that the observed symptoms were the result of alterations in normal hematopoiesis [1]. Not long after, Ernst Neumann identified leukemia as a disease originating in the bone marrow [3]. However, almost 100 years passed before new insight into the disease was gained. Only in 1960 did Peter Nowell and David Hungerford, after improving a method of visualizing chromosomes in mitotic cells (karyotyping), report the identification of an abnormal “minute chromosome” in patients with chronic granulocytic leukemia [4], representing the first discovery of a link between chromosomes and cancer. This chromosome later became known as the “Philadelphia (Ph) chromosome”. In 1973, Janet Rowley, while using chromosomal staining methods such as quinacrine fluorescence and Giemsa banding, observed that the minute chromosome was the result of the reciprocal translocation between chromosomes 9 and 22 (t(9;22)) [5]. This was the first demonstration of a chromosomal translocation being causative of a cancer. This translocation usually occurs in hematopoietic stem cells (HSC), which lie at the top of the hematopoietic hierarchy (Figure 1A). A few years later, Nora Heisterkamp and Jim Groffen showed that the human homolog of the Abelson murine leukemia viral oncogene (v-Abl), *ABL1*, was located in the region of chromosome 9, which translocates to chromosome 22 [6,7].

The translocation of the *ABL1* proto-oncogene was detected only in patients positive for the Ph chromosome [8]. Further, a small region of up to 5.8 kb on chromosome 22 was identified, within which all of the translocation breakpoints were occurring and which was named “breakpoint cluster region” (BCR) [9]. *BCR* later became the denomination of the gene on chromosome 22 fusing with *ABL1*. The majority of CML cases are due to a *BCR-ABL1* breakpoint, which fuses exons 13 or 14 of *BCR* with exon 2 of *ABL1* (called b2a2 or b3a2, respectively), leading to a BCR-ABL1 oncoprotein of 210 kDa (p210) [10]. Subsequent studies demonstrated the detection of a chimeric mRNA resulting from the fusion of the two genes, *BCR* and *ABL1*, in patients with CML [11,12]. In the same years, Konopka and colleagues observed the presence of an altered form of the c-ABL protein (p210) with tyrosine kinase activity in the Ph^+^ human leukemia cell line K562 [13]. This protein was shown to be the product of the fusion gene resulting from the t(9;22) translocation [14] leading to the cellular transformation of hematopoietic cells [15,16]. In confirmation of this, retroviral transduction of murine bone marrow with the *BCR-ABL1* fusion led to a CML-like myeloproliferative disorder in mice [17], proving the oncogenic capacity of this fusion protein and its central role in CML.

In summary, *BCR-ABL1* is the central player in the pathogenesis of CML, with the expression of its oncoprotein leading to clonal expansion of those hematopoietic cells, which harbor this fusion gene [18]. CML and its associated discoveries have acted as a paradigm for many cancers.

## 2. Characteristics of CML

CML belongs to the group of myeloproliferative neoplasms (MPN) characterized by the uncontrolled growth of myeloid cells at different stages of maturation. Patients may present in three disease phases: chronic phase (CP), accelerated phase (AP) and blast phase (BP) or blast crisis (BC), which is characterized by an increasing percentage of blasts of the myeloid, lymphoid or mixed/undifferentiated lineage, with myeloid BC occurring at 70%, approximately twice as frequently as lymphoid BC. According to criteria by the World Health Organization, 10–19% blasts in peripheral blood or bone marrow are counted as AP, while > 20% blasts are considered a criterion for BC [19] (Figure 1). Most of the patients present in CP, but, if left untreated, they will progress to AP and then to BC. A small fraction of patients may evolve directly to BC. The symptoms of CML are unspecific and may include fever, fatigue and weight loss, often as a result of anemia and splenomegaly. With progression to BC, the symptoms may become more severe and may include bone pain and bleeding. However, half of the patients in CP CML are symptom-free and may only be diagnosed after routine blood tests.

## 3. CML Stem Cells

Leukemia stem cells (LSC), including CML stem cells, are defined as the population of leukemia cells which give rise to the disease when transplanted into immunodeficient recipient animals [20] or sustain survival and self-renewal in optimized ex vivo culture [21] (Figure 1B). They are also believed to be the source of resistance to therapy and relapse. Single cell analysis of LSC from patients with CML revealed the existence of subgroups of LSC, such as LSC after long-term therapy and BC LSC, which can be distinguished by specific signatures. The identification of the different LSC subgroups may allow predictions on which patients will develop resistance or progress to BC [22]. In CP CML, LSC are thought to originate in the CD34^+^ CD38^−^ fraction of HSC [20,21] or from the primitive hemangioblast, whilst in BP CML and acute myeloid leukemia LSC may arise in HSC or in more committed cells of the hematopoietic hierarchy [21] (Figure 1A). The expression of certain markers characterizes CML LSC compared to their normal counterpart, the HSC, although this expression may be specific for the disease phase. These include:

- (a) the sialic acid receptor CD33, which was expressed in CP LSC but only variably in BP LSC [23],
- (b) the scavenger receptor CD36, expressed on primitive CML cells with decreased imatinib sensitivity [24] and on BC LSC in proximity to adipose tissue, whereby it mediates fatty acid uptake and oxidation [25],
- (c) the dipeptidylpeptidase IV CD26, which cleaves stromal-derived factor (SDF)1-α and, thereby, impairs the SDF-1α- C-X-C chemokine receptor type (CXCR)4-axis leading to an altered response to tyrosine kinase inhibitors (TKIs) [26,27],
- (d) the lectin transmembrane receptor CD93, which labels a population with increased stem cell characteristics, robust engraftment in xenotransplantation models and correlation with relapse upon TKI withdrawal [28],
- (e) the interleukin-1 receptor accessory protein (IL1RAP) [27,29],
- (f) the interleukin-2 receptor α CD25 [30] and
- (g) the interleukin 3 receptor subunit (CD123), a known marker for acute myeloid leukemia stem cells, but also CP and BP LSC [31].

CML LSC may also be identified by increased expression/activity of factors involved in self-renewal and/or drug resistance. β-catenin plays a key role in LSC from patients with CML in AP or BP by enhancing both their self-renewal and leukemia-inducing ability [18]. β-catenin also mediates the activation of pro-survival pathways in murine CML LSC, which are insensitive to imatinib treatment [32]. LSC resistant to TKI treatment have been shown to sustain their survival through the activation of the Janus kinase 2 (JAK2)/protein phosphatase 2A (PP2A)/β-catenin signaling pathway [33]. Proteomic and transcriptomic analysis of LSC from patients with CML revealed c-Myc and p53 as master regulators of a set of transcripts/proteins that were found altered in these samples. The stabilization of p53 and concomitant downregulation of c-Myc led to an increase in apoptosis and differentiation of LSC. The dual inhibition of c-Myc and p53 had a superior effect in inducing apoptosis in CML cells, while no significant effect was observed for normal CD34^+^ cells [34]. In addition, the zinc finger transcription factor Krüppel-like factor 4 (KLF4) maintains leukemia-initiating cells’ (LIC) survival and self-renewal capacity via downregulation of the dual-specificity tyrosine-(Y)-phosphorylation-regulated kinase 2 (DYRK2), which in turn reduces c-Myc protein levels and activates p53. The inhibition of the degradation of DYRK2 by using vitamin K3 leads to increased apoptosis and reduced self-renewal of murine and human CML stem/progenitor cells. This suggests that the regulation of DYRK-2 may represent a potential approach to hinder CML progression [35].

CML stem cells rely more on oxidative respiration than differentiated BCR-ABL1^+^ cells. Using stable isotope-mediated metabolic profiling of CD34^+^ and CD34^+^ CD38^−^ CML stem cell fractions and differentiated cells (CD34^−^), a dependency of CML cells on mitochondrial oxidative phosphorylation was confirmed. Combined treatment with imatinib and tigecycline, an antibiotic, which inhibits the synthesis of bacterial proteins, including mitochondrial proteins, led to the eradication of CML LSC in vitro and in vivo [36]. In BC CML, a sub-population of LSC has been observed to reside in proximity of gonadal adipose tissue and rely on fatty acid oxidation for survival. These LSC show a distinct upregulation of inflammatory cytokines and factors such as adipose triglyceride lipase, which promotes lipolysis and the release of free fatty acids (FFAs) by adipocytes. FFA intake is mediated by CD36, which is associated with resistance to chemotherapy via the microenvironment of gonadal tissue [25].

Imatinib induces autophagy in the cell lines of BC CML, primary CML cells and BCR-ABL1^+^ myeloid precursor cells [37]. A study showed that autophagy related 7 (ATG7), a component of the LC3 conjugation complex, plays a key role in mediating autophagy in CML LSC. Knockdown of *ATG7* increased mitochondrial respiration and levels of mitochondrial reactive oxygen species (ROS), promoting the differentiation of leukemic progenitors into the erythroid lineage. Knockdown of *ATG7* also sensitized leukemic progenitors to imatinib treatment [38].

While much information has been gained on the nature of CML LSC, which are independent of the function of BCR-ABL1 [39,40], their eradication has remained largely elusive and the necessity of their eradication is somewhat controversial [41]—even in view of the novel concept of treatment-free remission (TFR), as discussed below.

## 4. Signaling in CML

BCR-ABL1 activates various downstream signaling pathways leading to (a) altered adhesion to stromal cells and the extracellular matrix (discussed in Section 10), (b) promotion of survival and (c) inhibition of apoptosis and, overall, to cellular transformation and the acquisition of self-renewal capacity (Figure 1B). This is achieved by its constitutively active tyrosine kinase activity as result of the inhibition of the negative regulator domain SH3 by first exon *BCR* sequences [42]. The alteration of adhesion in BCR-ABL1-transformed cells [43,44] is largely due to Crk-like protein (CrkL), one of the tyrosine phosphorylated substrates of BCR-ABL1, as it is involved in cellular motility and adhesion mediated by integrins via its association with proteins of the focal adhesosome, such as paxillin, focal adhesion kinase (FAK) and others implicated in integrin signaling, including Cbl Proto-Oncogene (CBL), Crk-associated substrate (CAS) and human enhancer of filamentation (HEF1) [45]. In BCR-ABL1^+^ cells, paxillin, vinculin, p125FAK and the other focal adhesion proteins talin and tensin downstream of the integrins [46] are tyrosine phosphorylated.

BCR-ABL1 directly affects the cytoskeleton via its binding to F-actin with the C-terminal domain of ABL [47,48,49] and by regulating Rho-like GTPases, such as RHO, RAC, and CDC42 [50]. Via various intermediate proteins, BCR-ABL1 also upregulates the expression of certain adhesion molecules, such as CD44 [51,52] or integrin α6 [53], and stimulates the function of very late antigen (VLA)-4 and -5 in a BCR-ABL1 kinase-dependent manner [54]. In contrast, expression of the adhesion molecules L-selectin and P-selectin glycoprotein ligand (PSGL)-1 was decreased on murine BCR-ABL1^+^ LIC [55].

Three main signaling pathways have been linked to the constitutive activation of BCR-ABL1: (1) signal transducer and activator of transcription 5 (Stat5), (2) Ras/mitogen-activated protein kinase (MAPK) and (3) phosphoinositide 3-kinase (PI3K)/AKT mediated signaling [56]. Phosphorylation of STAT5 mediates the transcription of several pro-survival and pro-proliferative factors [57,58,59,60,61], as well as anti-apoptotic factors such as B-cell lymphoma-extra large (Bcl-xL) [62]. Similarly, PI3K/AKT signaling has been shown to have a key role in CML, mediating both the activation of cell survival [63] and possibly anti-apoptotic signaling [64], whereas the activation of Ras/MAPK signaling has been implicated in BCR-ABL1-dependent cellular transformation [65]. p27, a nuclear cell cycle inhibitor, is also targeted by BCR-ABL1 at multiple levels, leading to the promotion of cell-cycle progression. Interestingly, BCR-ABL1 activity not only blocks p27 by either inhibiting its transcription or promoting its degradation [66], but also promotes its cytoplasmic localization, where it has been described to promote oncogenesis [67]. BCR-ABL1 has been proposed to negatively regulate ABL1, which seems to have pro-apoptotic function [68]. Overall, BCR-ABL1 is a promiscuous signaling protein interfering with several aspects of the cellular machinery.

## 5. Additional Molecular Alterations beyond BCR-ABL1

The presence of the BCR-ABL1 oncoprotein is known to contribute to the acquisition of additional genetic abnormalities, likely by increased genomic instability [69]. This process of genetic diversification, clonal expansion and selection, termed clonal evolution, is associated with the advancement of the disease to BC, as well as an increased incidence of relapse, poor prognosis and resistance to TKI treatment, as described below [70,71,72]. Well known additional genetic abnormalities frequently found in BC are trisomy 8, isochromosome 17, duplication of the Ph chromosome or chromosome 19, 21 or 17, loss of chromosome Y or monosomy 7 [73,74]. Alterations, and particularly the loss of p53 or the loss of p16*INK4A/ARF* (the latter mostly in lymphoid BC), have also been identified in BC [75,76]. The loss of p53 is associated with increased resistance to apoptosis [77]. Recently, mutations in the polycomb repressive complex (PRC) pathway were discovered in the BC genome, with PRC2 directing DNA hypermethylation and silencing genes involved in myeloid differentiation and with tumor suppressor function and PRC1 repressing similar and novel tumor suppressors [78]. Other BCR-ABL1-independent, somatic mutations may also occur, especially in the genes *ASXL1, DNMT3A, RUNX1* and *TET2*, whereby these may also have been present prior to the *BCR-ABL1* rearrangement [79] and may represent clonal hematopoiesis in aged individuals [80]. In BC, an increase in the expression of pro-tumorigenic factors such as the Src family member Fyn, which may add to BCR-ABL1-induced genomic instability [81,82], B-cell lymphoma 2 (BCL-2) or c-Myc [83] has been observed. The latter has been shown to regulate the expression of several anti-apoptotic factors [84], to inhibit differentiation [85,86] and to possibly cause genomic instability [87]. From this short summary, it becomes obvious that our understanding of BC is far from complete. This is at least partly due to the lack of cell lines and murine models of BC CML. Future efforts must be directed at understanding the progression of CP CML on a molecular level.

## 6. Diagnosis of CML

The diagnosis of CML is made by pathology, cytogenetics and the detection of the *BCR-ABL1* transcript by reverse transcriptase-polymerase chain reaction (RT-PCR), or of the Ph chromosome by fluorescence in situ hybridization (FISH) [88]. RT-PCR is also used for following response to treatment by the assessment of molecular response (MR), which is defined as the ratio of *BCR-ABL1* to *ABL1* transcripts according to the International Scale (IS). An MR ≤ 1% is considered a complete cytogenetic remission, while an MR ≤ 0.1% indicates major molecular response (MMR) or MR^3^. Molecularly undetectable leukemia is defined as *BCR-ABL1* transcripts ≤ 0.0032% or MR^4.5^ [88].

## 7. Treatment of CML

Early treatments of CML at the beginning of the 1900s consisted of arsenic, radiotherapy of the spleen [89], busulfan, an alkylating agent [90], or the ribonucleotide reductase inhibitor hydroxyurea [91]. While the latter two treatments could result in a hematological remission, achieving a complete cytogenetic response by the elimination of Ph^+^ cells in the bone marrow of patients with CML was a rare event. The first drug capable of increasing hematological remission and partial or complete cytogenetic response was IFN α [92,93,94], which led to an improved long-term prognosis [94,95]. Later, in the majority of cases, HSC transplantation was shown to be successful at eliminating Ph^+^ cells, first when the donor HSC were obtained from an identical twin [96] and later from human leukocyte antigen (HLA)-matched siblings [97,98] or HLA-matched allogeneic donors. By the direct transplantation of non-T cell-depleted allografts or by the infusion of lymphocytes from the HSC donors, it became clear that it was the occurrence of the so-called graft-versus-leukemia (GvL)-effect which was largely responsible for the benefit of allogeneic HSC transplantation in CML [99,100]. Furthermore, the concept that leukemia cells could be targeted by antigen-specific T cells paved the way for immunotherapy in CML. Hereby, the BCR-ABL1 fusion, proteinase 3 and the Wilms tumor gene were considered promising immunological targets [101]. In further support of the role of CML as a “model disease”, the sophistication of its treatment demonstrating a powerful GvL-effect and culminating in targeted treatment of its oncogenic lesion in the early 2000s has been a success story, although setbacks were frequently encountered, as described in the following.

### Targeted Therapy of CML and Resistance

In 1996, the treatment of CML and, eventually, other malignant diseases was revolutionized by the advent of the compound CPG57148, a TKI of the 2-phenyl-aminopyrimidine class, called signal transduction inhibitor (STI) 571 and later imatinib mesylate, which inhibits the protein tyrosine kinase of ABL [102,103,104]. In a 10-year follow-up trial of CML patients on imatinib, it was shown that 83% achieved a complete cytogenetic response [105]. While the success of targeted therapy of CML with imatinib was unprecedented, complications in the form of primary and secondary BCR-ABL1-dependent and -independent resistance to imatinib arose. BCR-ABL1-independent mechanisms of resistance refer to factors such as the bioavailability of the drug, e.g., influx or efflux into or out of the cells, distribution and catabolism or alternative signaling pathways. BCR-ABL1-dependent mechanisms comprise increased expression of BCR-ABL1 or mutations in the *BCR-ABL1* kinase domain [106]. Accordingly, new, improved, second and third generation TKIs, such as nilotinib [107], dasatinib [108] and bosutinib [109], were developed. Of the newer agents, the third line TKI ponatinib was specifically developed to target the *BCR-ABL1^T315I^* and compound mutations [110], while asciminib (ABL001), an allosteric inhibitor of the ABL-kinase, exerts its effect by binding to the myristoyl and not the catalytic pocket of BCR-ABL1 [111]. These agents and mechanisms of resistance are reviewed in detail elsewhere.

## 8. Treatment Free Remission

A recent study suggests that the life expectancy of CML patients treated with TKIs is close to the life expectancy of the general population [88,112]. Lifelong treatment with TKIs for CML was recommended until recently. However, given off-target effects, adverse events, toxicities of TKIs [113,114] and the high costs associated with lifelong treatment with TKIs, scientists and clinicians are now exploring the possibility of discontinuing TKI treatment in those patients who have achieved a deep molecular remission (*BCR-ABL1* < 0.01). This may represent a risky undertaking, as the persistence of LSC post TKI treatment has been extensively documented in various in vitro and in vivo models [21,115,116,117]. It has been shown that CML stem cells do not depend on BCR-ABL1 activity and that targeting BCR-ABL1 will not eliminate CML stem cells [39]. Therefore, it is crucial to identify those patients who may remain in treatment-free remission (TFR) and to understand which biological factors may or may not maintain TFR. In early trials on the discontinuation of imatinib, 40–60% of patients remained in complete molecular remission after discontinuation of a TKI [118,119,120,121].

Considering the profound knowledge of CML, which has accumulated over the years, the concept of TFR is relatively new, and little is known about the molecular components regulating TFR.

One study showed that TFR may be influenced by the type of *BCR-ABL1* transcript. Patients with the e13a2 transcript have reduced deep molecular remissions, and only 3% obtained durable TFR compared to patients with the e14a2 transcript [122]. In addition, the combination of a TKI with IFN α and subsequent IFN α as a maintenance therapy may allow TKI discontinuation [123].

A few studies point out the involvement of immune cells for the control of TFR. It has been demonstrated that there is an increase in natural killer (NK) cells and a decrease in CD3^+^ CD8^+^ CD62L^+^ cells in patients in whom imatinib was discontinued (STOP-IM) compared to CML patients taking imatinib in complete molecular remission (CMR), which was positively associated with CMR [124]. Another trial (EURO-SKI) revealed a higher rate of TFR in CML patients with a higher percentage of NK, but not B- or T-cells. These NK cells were mature compared to relapsing patients, in whom NK cells showed a more naïve phenotype. Additionally, increased tumor necrosis factor (TNF) α/IFN γ secreted by NK cells was correlated with the achievement of TFR [125]. Additionally, expression of the T-cell inhibitory receptor (CTLA-4)- ligand CD86 on plasmacytoid dendritic cells (pDC) also predicted the TFR rate, as found in EURO-SKI trial patients. Patients with <95 CD86^+^ pDC per 10^5^ lymphocytes showed an increased one-year relapse- free survival compared to patients having >95% CD86^+^ pDC. This was found to be due to exhaustion of leukemia-specific CD8^+^ T cells. Additionally, patients with <95 CD86^+^ pDC per 10^5^ lymphocytes experienced a significant benefit from extended TKI treatment before discontinuation [126]. In another multicenter study, D-STOP, patients with a lower percentage of CD3^−^ CD56^+^ or CD16^+^ CD56^+^ NK or CD56^+^ CD57^+^ NK-large granular lymphocytes (LGL) cells obtained longer treatment-free survival post consolidation of dasatinib therapy [127]. Increased NK cells with higher expression of NK activating receptors, but decreased levels of suppressive FoxP3^+^ regulatory T cells and monocytic myeloid-derived suppressor cells, were found in patients achieving TFR [128]. In contrast, in the STAT2 trial, no differences in treatment-free survival were observed with regards to absolute numbers of NK cells [129]. The exciting concept of TFR, which is rarely successful in other cancers, is currently the object of intense study in the CML field. The discovered contributions of the immune system to TFR highlight the importance and still largely unknown role of the immunological microenvironment in the bone marrow and other organs for CML and, probably, other hematological malignancies. More insight in this area may be expected in the near future.

## 9. Metabolic Targeting in CML

Altered metabolic demands of cancer cells have been reported for various malignancies, including CML. These might serve as potential targets in the future. A cytosolic enzyme, branched chain amino acid transferase (BCAT)1, was found to be upregulated in humans and murine models of CML, with its amount increasing with disease progression. Targeting the gene expression or activity of BCAT1 induces cellular differentiation and inhibits BC progression. Musashi2 (MSI2), an RNA binding protein, binds and stabilizes BCAT1 RNA and, thereby, promotes CML [130]. Another study compared the global metabolic differences between murine HSC and CML stem cells, finding that CML stem cells accumulate significantly higher amounts of certain dipeptide species, which, when internalized, can activate p38MAPK and Smad3, regulating amino acid signaling and CML stemness. Pharmacological inhibition of the uptake of dipeptides compromised CML stem cell activity by targeting Smad3 signaling [131].

The nicotinamide adenine dinucleotide (NAD)-dependent histone deacetylase sirtuin 1, SIRT1, which promotes mitochondrial respiration via the deacetylation and activation of the peroxisome proliferator-activated receptor γ coactivator 1α (PGC-1α), a regulator of mitochondrial biogenesis, has been shown to be activated in BCR-ABL1-transformed cells [132,133]. SIRT1 activation via STAT5 signaling resulted in the deacetylation of several substrates promoting cell survival and proliferation. The inhibition or knockdown of *SIRT1* induced apoptosis in CML LSC via p53 [133] and impeded CML induction by reducing the expression of mitochondrial genes in CML stem and progenitor cells, while the combination of a TKI with *Sirt1* deletion further suppressed CML progression [134]. Furthermore, single cell transcriptome data of CML patient-derived versus normal HSC samples showed an upregulation of genes associated with oxidative phosphorylation and glycolysis [22]. The absence of either of the glycolytic enzymes pyruvate kinase isoform M2 (PKM2) or lactate dehydrogenase A (LDHA) significantly delayed the onset of CML-like disease in mice [135]. PKM2 was found to be increased in TKI-resistant primary cells and cell lines. Knockdown of *PKM2* reduced growth and increased apoptosis in these cell lines after treatment with imatinib [136]. Despite a few potential targets, the field of metabolism in cancer and particularly the targeting of metabolic pathways is young, and it will need to be investigated in how far in vitro experiments, mostly performed under stable conditions, realistically mirror the physico-chemical complexities in a tumor or the bone marrow microenvironment (BMM) impacting a cancer’s metabolism. In addition, leukemia-induced metabolic alterations in the BMM may perturb the homeostasis of normal hematopoietic cells, thereby adding to the impairment of normal hematopoiesis in the presence of leukemia.

## 10. The Role of the Bone Marrow Microenvironment in CML

The interdependencies of leukemia cells and their BMM are becoming increasingly evident. Leukemia cells are known to interact with, alter and exploit their surrounding niche, in order to render it more permissible for leukemia progression. However, these same factors, molecules, and mechanisms that mediate leukemia–BMM interactions might represent a vulnerability of the disease and serve as a potential target for treatment [115,137]. The BMM, for example, is hypoxic, with the lowest oxygen tension being found deep in the perisinusoidal regions [138]. The complicated interplay between the permeability of arterioles and levels of ROS—despite the increased vascular density found in hematological malignancies [115]—influences metabolism in leukemia cells, as well as their expression of hypoxia-inducible factor-1α (HIF1α), which has been found to be important for CML progression [139].

**Figure 2:**
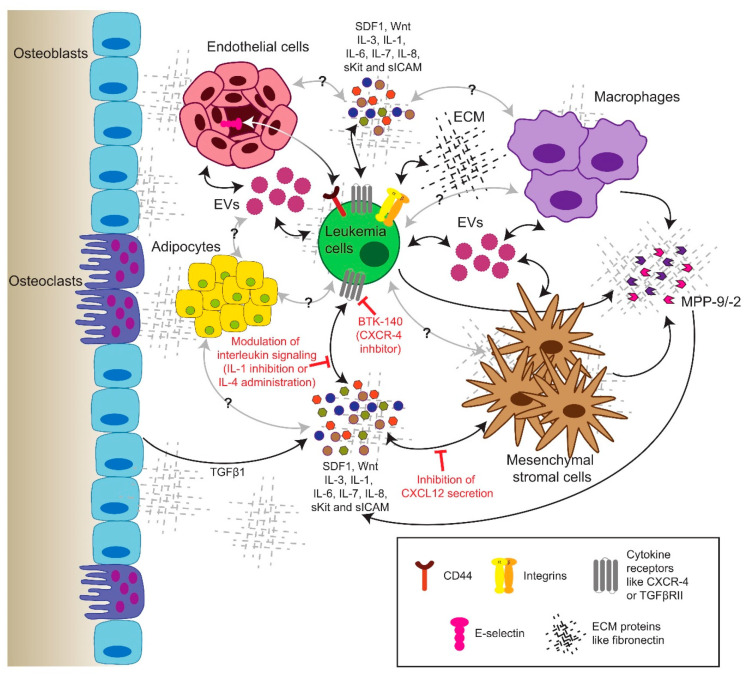
Schematic delineating the anatomy of the bone marrow microenvironment of CML (stem) cells, as well as their interactions with different niche cell types, secreted factors (whose variety is represented by different shapes and colors) and components of the extracellular matrix. Different strategies for targeting these interactions between CML stem cells and their microenvironment in combination therapies are presented in red. The black arrows represent interactions which are known, while the gray arrows represent possible interactions which have not yet been described. EVs = extracellular vesicles; SDF-1 = stromal-derived factor 1 (= C-X-C motif chemokine 12 (CXCL12)); IL = interleukin; ECM = extracellular matrix; sKit = soluble Kit; sICAM = soluble intercellular adhesion molecule; MMP-2/9 = matrix metalloproteinase-2/-9; TGFβ1 = transforming growth factor β 1; TGFβRII = transforming growth factor receptor II; CXCR-4 = C-X-C chemokine receptor type 4.

CML cells interact with their BMM via specific pathways. In fact, CML progenitors adhere less to stroma and the extracellular matrix protein fibronectin via integrin β1 [140]. However, this may be restored by treatment with IFNα [141]. Furthermore, the integrin-mediated adhesion of leukemia cells to fibronectin leads to proteasomal degradation of the pro-apoptotic protein Bim (BCL2 like 11), promoting cell survival and contributing to minimal residual disease and chemoresistance [142]. CML cells resistant to imatinib due to the *BCR-ABL1^T315I^* mutation exhibited increased expression of integrin β3 and integrin-linked kinase (ILK), a component of the focal adhesosome, as well as altered niche localization compared to *BCR-ABL1^WT^* cells. The modulation of the extracellular matrix by the alteration of fibronectin levels prolonged disease progression [143] (Figure 2). ILK was also shown to be a critical factor for the pathogenesis of CML and the maintenance of quiescent stem cells in the presence of TKIs [144].

Further, reduced β1 integrin avidity also affects the function of the adhesion molecule CD44, thereby contributing to the circulation and expansion of CML progenitors [145]. In fact, CD44 or homing cell adhesion molecule (HCAM), a cell membrane glycoprotein, which interacts with different BMM-associated proteins such as hyaluronan, osteopontin or E-selectin, was shown to be crucial for CML and the engraftment of CML cells [51]. In addition, CML-initiating cells rely to a greater extent on selectins and their ligands for homing and engraftment than do normal stem cells [55]. The blockade of adhesion of CML-initiating cells to E-selectin expressed on the bone marrow endothelium increased cell cycle progression and the SCL/TAL1 signaling axis in leukemia cells, improving the efficacy of imatinib treatment [52]. In the presence of imatinib, CML cells upregulate the expression of C-X-C chemokine receptor type 4 (CXCR-4), promoting the migration of leukemia cells to bone marrow niches and their chemoresistance in response to stimulation with stromal-derived factor (SDF)1 [146]. The combination of the CXCR4 inhibitor BTK140 with imatinib showed a synergistic effect in the eradication of leukemia cells [147]. Another study showed that the inhibition of CXCR4 with plerixafor increased leukemia cell mobilization and sensitivity towards nilotinib [148], but in a different, aggressive murine model, treatment with dasatinib and plerixafor was not superior to dasatinib alone [149]. Low levels of CXCL12 (=SDF1), a regulator of LSC homing to the bone marrow and of their quiescence, have been detected in both mice and patients with CML [150]. Concordantly, a recent study suggested that the targeted depletion of CXCL12 specifically from mesenchymal stromal cells (MSC) promoted CML LSC self-renewal, including in the presence of a TKI, while reducing the number of normal HSC. In contrast, the endothelial cell-specific ablation of CXCL12 decreased LSC proliferation [151] (Figure 2).

### 10.1. Soluble Factors in the Extracellular Milieu

Interleukins (IL), known to be secreted by different niche cell types such as MSCs, endothelial cells, but also leukemia cells themselves, represent important soluble factors in the BMM, which are able to regulate CML progression. BCR-ABL1-dependent IL-6 expression by CML cells, for example, led to the establishment of a proinflammatory tumor environment and—via an autocrine activation loop—sustained CML development [152]. Additionally, normal mouse hematopoietic progenitor cells in the BMM of mice with CML acquired a gene expression pattern similar to CML cells and exhibited faster division, altered differentiation and reduced reconstitution and self-renewal potential. This phenomenon was largely mediated by IL-6, as the inhibition of IL-6 changed the gene expression of normal hematopoietic stem and progenitor cells and their lineage bias, thus counteracting the progression of the disease [153].

The suppression of IL-1 using soluble IL-1 or receptor antagonists reduces the colony growth of CML cells [154]. Recent findings further showed the efficacy of combining IL-1 inhibition with imatinib to eliminate LSC [29]. In contrast, the anti-proliferative effect of IL-4 was shown on the colony growth of CML cells [155]. Another study revealed the importance of IL-7 secreted by MSCs in promoting resistance to TKIs [156]. IL-3 can protect BCR-ABL1-transformed hematopoietic progenitors from TKI-induced apoptosis [157].

Although the low-level autocrine secretion of transforming growth factor (TGF)-β1 by CML LSC regulates AKT activation and the nuclear localization of forkhead box protein O3 (FOXO3A), thereby maintaining LSC [158], high levels of TGF-β1 released from the extracellular matrix of an actively remodeling BMM have an inhibitory effect on the progression of CML [159] (Figure 2). Additionally, placenta-derived growth factor (PlGF) is increased in CML, stimulating angiogenesis in the bone marrow and promoting CML cell proliferation, metabolism and disease progression, while the inhibition of PlGF synergizes with imatinib [160].

### 10.2. Extracellular Vesicles

Exosomes are small extracellular vesicles (30–150nm) that are secreted upon the fusion of the multivesicular bodies with the plasma membrane. They play an important role in intercellular communication in different cancers [161]. CML-derived exosomes have been shown to be able to remodel the BMM by inducing neovascularization [162] and stimulating the production of IL-8, which in turn modulates the malignant leukemic phenotype [163]. Exosomes can mediate the exchange of several types of molecules, including miRNA. Accordingly, it has been demonstrated that the exosome-mediated transfer of miR-320 to stromal cells inhibited osteogenesis by MSC and, hence, the remodeling of the BMM, thereby influencing leukemia progression [164].

Taken together, the role of the BMM in hematological malignancies and CML in particular is a complex and challenging field. Future efforts must be directed at obtaining patient CML samples in the form of bone marrow biopsies and optimizing strategies of culturing specific niche cells, such as endothelial or osteoblastic cells, or designing artificial niches in order to augment our understanding of the BMM and pave the way for its successful targeting in combination therapies.

## 11. Molecular Targets beyond TKI and Combination Treatments

### 11.1. Targeting of Alternative Signaling Pathways

The hedgehog (Hh) signaling pathway regulates cell proliferation and response to stress and injury, while also maintaining stem cells. It involves the binding of an HH ligand, such as sonic hedgehog, to the receptor Patched (Ptch-1), which releases the smoothened (Smo)-mediated repression, leading to the nuclear translocation and activation of the glioma-associated oncogene (Gli) transcription factors [165]. While HH signaling during adult hematopoiesis is dispensable [166], it seems to play an important role in the expansion of LSC in CML. The inhibition of Smo leads to a reduction in LSC and increased relapse-free survival in an in vivo model [167]. Gene and protein expression of HH pathway members were found to be activated in a murine model of CP CML. Treatment with sonidegib, a SMO inhibitor, and nilotinib reduced the colony- forming ability of CD34^+^ CP CML cells. The treatment also inhibited the engraftment of human CML LSC in the xenotransplantation system and reduced leukemia development in secondary recipients of mice treated with the combination therapy [168]. An upregulation of autophagy was observed in CML cells treated with vismodegib, an HH pathway inhibitor. The combination of vismodegib and autophagy inhibitors or the silencing of *ATG5* and *ATG7* significantly reduced cell viability and significantly enhanced CML cell death [169].

**Figure 3:**
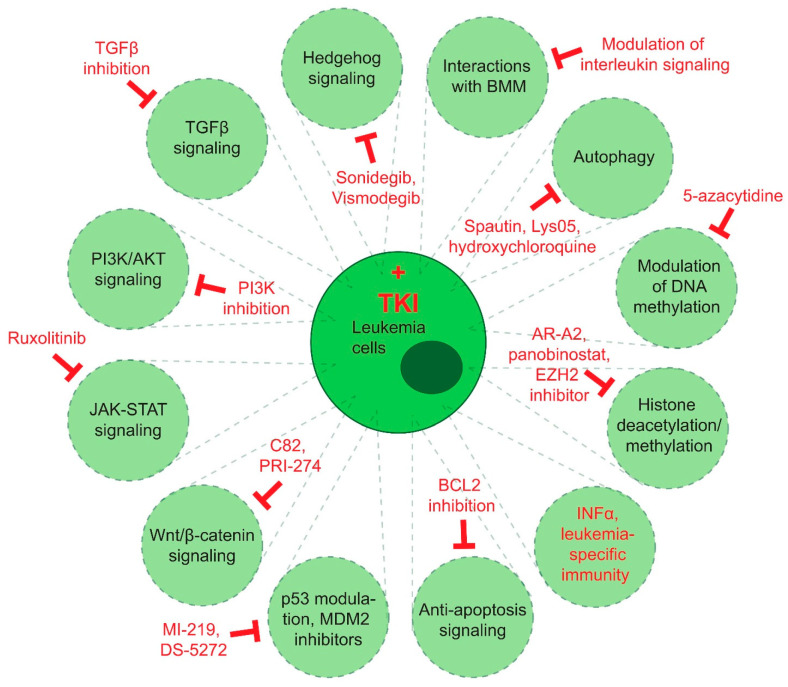
Strategies for targeting CML stem cells (in red) with tyrosine kinase inhibitors (TKIs) and other agents in combination therapies. TGFβ1 = transforming growth factor β 1; EZH2 = Enhancer of zeste homolog 2; BCL-2 = B-cell lymphoma ***2***; PI3K/AKT = phosphoinositide 3-kinase/AKT Serine/Threonine Kinase 1; IFNα = interferon α; MDM2 = MDM2 proto-oncogene; JAK-STAT = Janus kinase (*JAK*)-signal transducer and activator of transcription.

The PI3K/AKT/mTOR signaling network functions downstream of multiple receptors and growth factor signaling pathways, representing a signaling hub for oncogenic signaling [170]. The inhibition of PI3K or mTOR signaling pathways in cell lines and patient-derived CML stem and progenitor cells increased sensitization to nilotinib and enhanced apoptosis [171]. In CML patient samples, the microRNA (miR)-21 level was found to be upregulated in patients in whom the TKI response was not optimal [172]. Consistently, other work showed that silencing miR-21 by antagomiR-21 or PI3K inhibition in combination with imatinib decreases AKT phosphorylation and MYC expression, suggesting that miR-21 mediates its effect by regulating the PI3K/AKT axis [173] (Figure 3).

Gene expression profiling of CD34^+^ Lin^−^ cells from CML patients after 12 months of nilotinib treatment showed downregulation of genes associated with JAK-STAT signaling [174]. A combination of the JAK2 inhibitor ruxolitinib with nilotinib led to decreased leukemogenic activity and the engraftment of CML stem and progenitor cells [175]. Targeting the BCR-ABL1-JAK2 complex with imatinib and a selective JAK2 inhibitor increased apoptosis, also of imatinib-resistant cells, and decreased the leukemia-initiating properties of CD34^+^ leukemic progenitor cells in xenotransplantation experiments [176]. Different clinical trials employing the JAK2 inhibitor ruxolitinib have been initiated. Ruxolitinb was tested in combination with nilotinib in CML patients with molecular disease (NCT01702064) or CML and Ph^+^ acute lymphoblastic leukemia patients (NCT02253277). Furthermore, IL-6 was shown to activate JAK1-STAT3 signaling in CML LSC and co-inhibition of JAK1 and BCR-ABL1 reduced the colony forming ability of murine and human CML cells, even in quiescent cells [177]. Modulation of the Wnt/β-catenin signaling pathway by C82 or its prodrug PRI-274 and nilotinib led to the killing of imatinib-resistant BC-CML cells in vitro and prolonged survival in xenotransplantation models [178].

The stabilization of p53 using the inhibitor MI-219, which targets the interaction of p53 with its inhibitor mouse double minute 2 homolog (MDM2), induces apoptosis in CML BC cells, irrespective of T315I mutation status. This effect was mediated by downregulation of c-MYC and upregulation of p21. In addition, the pro-apoptotic proteins PUMA, NOXA, BAX and CD95/Fas were also activated, suggesting the involvement of both intrinsic and extrinsic apoptosis pathways [77]. Similarly, given increased p53 signaling in CML cells, treatment with a TKI and the inhibitor of MDM2, DS-5272, leads to increased levels of NOXA, while decreasing leukemia burden and LSC and prolonging survival in a murine model of CML [179].

Expression of BCL2, a regulator of apoptosis, is increased in CML LSC, and the inhibition of BCL2 in combination with a TKI reduced the number of LSC in samples from patients with CP and BC CML and significantly prolonged survival in a murine model of CML [180].

The observation of hematologic remissions and complete cytogenetic remissions after single treatment with IFN 2α in 13 to 27% of cases [95] prompted studies on the combination of IFN 2α with imatinib with the intent of eradicating LSC by the stimulation of cytotoxic T cells [181]. It was also hypothesized that the simultaneous or sequential administration of imatinib and IFN 2α may allow the discontinuation of imatinib. A retrospective analysis of CML patients, for example, revealed a higher rate of complete cytogenetic remission after six months of treatment with IFN α plus imatinib compared to imatinib alone [182]. In another trial, 15 out of 20 patients on imatinib and IFN α remained in remission on IFN α almost 2.5 years after discontinuation of imatinib [183]. Molecular response was also higher in the SPIRIT trial in patients on imatinib and peg-interferon [184], but there have not been any effects on survival so far [185].

Possible strategies for the targeting of the BMM in conjunction with TKI will increasingly arise with the expansion of research in this area, but, currently, may be considered for the initiation of clinical trials on the use of antibodies to IL-6 [152,153] or PlGF [160], parathyroid hormone [159], E-selectin antagonists [52], fibronectin or inhibitors of integrin-linked kinase [143,144], as mentioned in the section on the BMM.

### 11.2. Targeting Epigenetic Modification

Histone deacetylases (HDAC) are a class of proteins that mediate the removal of acetylation from lysines on histones, rendering DNA more accessible for regulation [186]. Quiescent CML LSC have been shown to be efficiently targeted by HDAC inhibitors in combination with imatinib [187]. A similar synergistic effect was found between imatinib and AR-42, a pan-HDAC inhibitor [188], as well as between ponatinib and panobinostat, a pan HDAC inhibitor [189]. Various clinical trials in CML patients have been completed with panobinostat in combination with TKIs [189].

Differential methylation patterns have been shown to characterize the three phases of CML [190]. Downregulation of the pro-apoptotic BCL2-interacting mediator BIM by methylation is associated with reduced response to imatinib [191]. The alteration of epigenetic reprogramming via inhibition of enhancer of zeste homolog 2 (EZH2), a member of the polycomb repressive complex 2, increases CML LSC sensitivity to apoptosis. This effect is potentiated by the combination of an EZH2 inhibitor with a TKI, leading to efficient eradication of LSC in vitro and in vivo [192]. Targeted deletion of CXCL12 in MSC, as discussed above, also increased the EZH2 activity in LSC, leading to enhanced cell proliferation and self-renewal [146]. Indeed, not only the inhibition of histone methylation but also the modulation of DNA methylation might be a feasible target for CML, as multiple reports have shown that a combination of 5-azacytidine, which regulates DNA methylation, with TKI may be effective in targeting the disease, including BC CML [193,194].

### 11.3. Autophagy

Autophagy is an important catabolic survival pathway employed by LSC. Early studies reported that autophagy in BCR-ABL1 expressing cells leads to a reduced p53-mediated stress response, and hence a reduced pro-apoptotic signal. The treatment of BCR-ABL1^+^ cells with imatinib led to autophagy and, thereby, drug resistance in addition to endoplasmatic reticulum stress and a reduction in intracellular calcium. This suggests autophagy as a resistance mechanism, and its targeting via an inhibitor or deletion of autophagy-related genes in combination with imatinib increased cell death in primary CML cells [37]. The autophagy inhibitor, spautin, in combination with imatinib increased apoptosis of CML cells via inactivation of the PI3K/AKT pathway and downregulation of anti-apoptotic proteins [195].

Expression of the autophagy protein ATG4B differed in CML cells of patients who responded to imatinib and those that did not. Knockdown of *ATG4B* decreased the survival of CML stem and progenitor cells and sensitized them to imatinib [196]. It was further shown that leukemic long-term (LT)-HSC have a higher basal level of autophagy compared to non-leukemic LT-HSC, and that targeting autophagy using the second generation autophagy inhibitor Lys05 in addition to TKI reduces primary and xenografted LSC [197]. Finally, a randomized phase II trial testing the effects of the combination treatment of hydroxychloroquine and imatinib versus imatinib alone in CML patients with residual disease suggests a potential benefit in targeting autophagy in CP CML [198], but larger trials need to be performed.

Despite the myriad of potential targets for possible combinations of treatment strategies, most of the trials on combination treatments performed have not yet yielded a significant breakthrough, and single agent TKIs remain the treatment of choice for now. The lack of an impressive response to combination therapies may have been due to unexpected toxicities or ineffectiveness of these therapies, whereby the latter may be caused by differences in host (patho-) physiology between laboratory mice and humans and the general limitations of murine models, for instance, with regards to the immune system, pharmacokinetics and -dynamics. It is assuring that the field has not become complacent in view of the accomplishments made in CML research and remains focused on finetuning these shortcomings, working towards a more complete understanding of CML while aiming at the ultimate state of TFR.

## 12. Conclusions

In the outlined work, we have discussed the events from the first identification of CML as a disease to the development of the “magic bullet” against this malignancy, as well as the difficulties arising with TKIs such as the non-targeting of LSC or the acquisition of mutations leading to TKI resistance. As CML can reemerge after discontinuation of TKI-treatment despite the availability of various and more potent TKIs, our focus has now shifted to the possibility of TFR and the physiological factors and molecular pathways which may influence this condition. Combination therapies, such as the targeting of leukemia cell-intrinsic pathways such as metabolism, autophagy or epigenetics, or leukemia cell-extrinsic pathways such as the immunological landscape of a patient or the BMM, appear to be attractive strategies. With the increasing discovery of the involvement of further molecules and pathways, we believe the scientific community will—at some point—be able to achieve TFR in our patient population and, finally, target and eradicate CML once and for all.
